# Association of *IFN‐γ* polymorphisms with ankylosing spondylitis risk

**DOI:** 10.1111/jcmm.15680

**Published:** 2020-07-30

**Authors:** Yanhui Liu, Guohui Zhang, Yulong Guan, Xiaoliang Zhao, Quan Wang, Hua Li, Jinhong Qi

**Affiliations:** ^1^ Department of Orthopedics Harrison International Peace Hospital The People's Hospital of Hengshui City Hengshui China

**Keywords:** ankylosing spondylitis, *IFN‐γ*, polymorphisms

## Abstract

The case‐control study was designed to investigate the genetic effects of interferon‐gamma (*IFN‐γ*) rs2069727 and rs1861494 polymorphisms on ankylosing spondylitis (AS) susceptibility in a Chinese Han population. Blood samples were collected from 108 AS patients and 110 healthy controls. *IFN‐γ* polymorphisms were genotyped by polymerase chain reaction‐restriction fragment length polymorphism (PCR‐RFLP). Hardy‐Weinberg equilibrium (HWE) test was performed in control group. Odds ratios (OR) with 95% confidence intervals (95% CI) were calculated using chi‐square test to evaluate the association between AS susceptibility and *IFN‐γ* polymorphisms, and the results were adjusted by logistic regressive analysis. The frequency of rs2069727 CC genotype was much higher in cases than that in controls, suggested its significant association with increased AS risk (adjusted OR = 5.899, 95% CI = 1.563‐22.261; *P* = .009). In addition, C allele also showed close association with increased risk of AS (adjusted OR = 2.052, 95% CI = 1.286‐1.704, *P  *= 0 .003). While the genotype and allele frequencies of *IFN‐γ* rs1861494 polymorphism were not significantly different between patients and controls (*P  *> 0.05 for all), *IFN‐γ* rs2069727 polymorphism is significantly associated with increased AS risk in a Chinese Han Population.

## 1 INTRODUCTION

Ankylosing spondylitis (AS) is one of the most common chronic inflammatory disorders of spondyloarthropathy (SpA).^1^ AS mainly affects axial skeleton, sacroiliac joint and peripheral joints, its clinical manifestations include pain, stiffness and a progressive thoracolumbar kyphotic deformity.^2^, ^3^, ^4^ The morbidity of AS is high among young adults especially males, causing significant effects on labour ability and quality of life.^5^ AS treatment remains a great challenge due to its asymptomatic nature at early stages,^6^ in early stages, there are no specific symptoms, and many AS patients are diagnosed with advanced stages and miss the optimal time for treatments, leading to incapacity and decreased quality of life. Until now, the aetiology of AS can not be explained completely. It is generally considered that the initiation and development of AS are regulated by the interactions between genetic and environmental factors.^7^, ^8^ Human leucocyte antigen (HLA)‐B27 was the firstly identified genetic factor in aetiology of AS, and approximately 95% AS patients show positive to the gene.^9^ However, population studies have demonstrated that only 2% HLA‐B27–positive patients will finally develop AS, revealing that in addition to HLA‐B27 gene, some other genetic factors also take part in pathogenesis of AS.^10^ Therefore, it is necessary to explore novel AS‐related genes which will be of great help for prevention, diagnosis and treatment of AS in clinic.

Interferon‐*γ* (Interferon‐gamma, IFN‐γ or *IFNG*) is an important cytokine, which is mainly secreted by natural killer cells and CD80^+^ T cells.^11^ The human *IFN‐γ* gene is located on chromosome 12 (12q14), containing 4 exons and 3 introns with approximately 6 kb.^12^ As an immunomodulatory factors, *IFN‐γ* plays an important role in activation of macrophage, and abnormal expression of *IFN‐γ* has been reported to be associated with a variety of auto‐inflammatory and immune diseases, such as chronic periodontitis, IgA nephropathy and multiple sclerosis.^13^, ^14^, ^15^, ^16^ In addition, *IFN‐γ* could also regulate human mesenchymal stem cell (MSC) osteogenesis, and *IFN‐γ* exposure might suppress MSC osteogenesis.^17^ All the data revealed that the abnormal expression of *IFN‐γ* might contribute to pathogenesis of AS. In AS, a study carried out by Wang H et al reported that the protein expression level of IFN‐γ was significantly higher in AS patients than that in health individuals.^18^ The transcription activity of *IFN‐γ* may be regulated by its genetic mutations.^19^ A related case‐control study reported that *IFN‐γ* rs2430561 polymorphism may contribute to risk of AS via influencing *IFN‐γ* expression.^20^ Rs2069727 and rs1861494 are two common polymorphisms in *IFN‐γ* gene, and both of them have the possibility to influence production of IFN‐γ protein.^19^, ^21^ According to the previous researches, we hypothesized that *IFN‐γ r*s2069727 and rs1861494 polymorphisms might be involved in AS. However, the related studies had been rarely reported in Chinese Han population.

In this study, we compared the genotype distributions of the *IFN‐γ* gene rs2069727 and rs1861494 polymorphisms between AS patients and healthy controls. In addition, the genetic effects of *IFN‐γ* gene polymorphisms on AS susceptibility were also investigated among the study population.

## 2 MATERIALS AND METHODS

### 2.1 Subjects

A case‐control study was conducted in Harrison International Peace Hospital, the People's Hospital of Hengshui City. There were 108 AS patients as case group and 110 healthy individuals as control group. AS patients were diagnosed by X‐ray, computed tomography (CT) and magnetic resonance imaging (MRI). The controls were recruited from the healthy individuals who received a medical examination in the same hospital during the study period. The participants would be excluded if they had bone diseases, systemic diseases, and immune or inflammatory diseases.

This study was approved by the Research Ethics Committee of Harrison International Peace Hospital, the People's Hospital of Hengshui City. All subjects were informed the study objective and flow. Before sample collection, written consent was signed by each included subject.

### 2.2 DNA extraction

After fasting for 10 hours, 5 mL peripheral venous blood was collected from each participant using EDTA‐coated tubes. Genomic DNA was extracted from blood samples using TIANamp Genomic DNA Kit (Beijing, China) according to manufacturer's protocols and then stored at −20℃ for standby application.

### 2.3 Genotyping

Genotype distributions of *IFN‐γ* gene rs2069727 and rs1861494 polymorphisms were detected by polymerase chain reaction‐restriction fragment length polymorphism (PCR‐RFLP) method. Primer sequences for *IFN‐γ* gene rs2069727 and rs1861494 polymorphisms were designed by Primer Premier software and showed in Table 1. The PCR procedure was carried out according the following steps: 95℃ pre‐degeneration for 5 minutes, 30 cycles of degeneration at 95℃ for 30 seconds, 57℃ annealing for 30 seconds, 72℃ extension for 30 seconds and final extension at 72℃ for 5 minutes.

**TABLE 1:** Primer sequences of *IFN‐γ* gene two polymorphisms rs2069727 and rs1861494

SNP	Primer sequences	Annealing temperature (℃)	Digested enzyme	
rs2069727	Sense	5'‐AGGTTCTGCTATGGAATGTA‐3'	56	*Hinf*I
	Reverse	5'‐AAACTACATTCCATAGCAGA‐ 3'		
rs1861494	Sense	5'‐CCATTCGTGTTTGGGTGA‐3'	57	*Aci*I
	Reverse	5'‐GTGGCTGAGTTGGGAGGA‐3'		

Subsequently, the PCR products were digested by the corresponding restriction enzymes (*Hinf*I for rs2069727 and *Msp*I for rs1861494). Then, the enzyme digestion results were analysed by 2% agarose gel electrophoresis and visualized by UV light. In addition, 20% of the amplified specimens were randomly selected for direct sequencing to estimate the efficacy of digestion results.

### 2.4 Statistical analysis

All the data analysis was conducted using PASW Statistics 18.0 statistical software (SPSS Inc, Chicago, IL, USA). The statistic power of our study was estimated by GPower 3.1 software. Hardy‐Weinberg equilibrium (HWE) was tested in control group based on genotype distributions of the detected *IFN‐γ* polymorphisms. Chi‐square test was applied to compare the genotype and allele distributions of *IFN‐γ* gene polymorphisms between AS group and control group. The genetic effects of *IFN‐γ* gene polymorphisms on AS were estimated by odds ratios (OR) with 95% confidence intervals (CI), and the results were adjusted using logistic regression model. *P* value less than 0.05 was considered as statistically significant.

## 3 RESULTS

### 3.1 Statistic power and HWE test

The statistic power of our analysis was estimated by GPower 3.1 software. Analysis results suggested the static power for *IFN‐γ* rs2069727 analysis was 0.769, and for rs1861494 polymorphism was 0.478. Genotype, allele frequencies of both *IFN‐γ* rs2069727 and rs1861494 polymorphisms in control group conformed to HWE (*P* = 0.970 and 0.205, respectively), indicating that our study population had a good representativeness for the general population.

### 3.2 Distributions of IFN‐γ gene polymorphisms between groups

**FIGURE 1:**
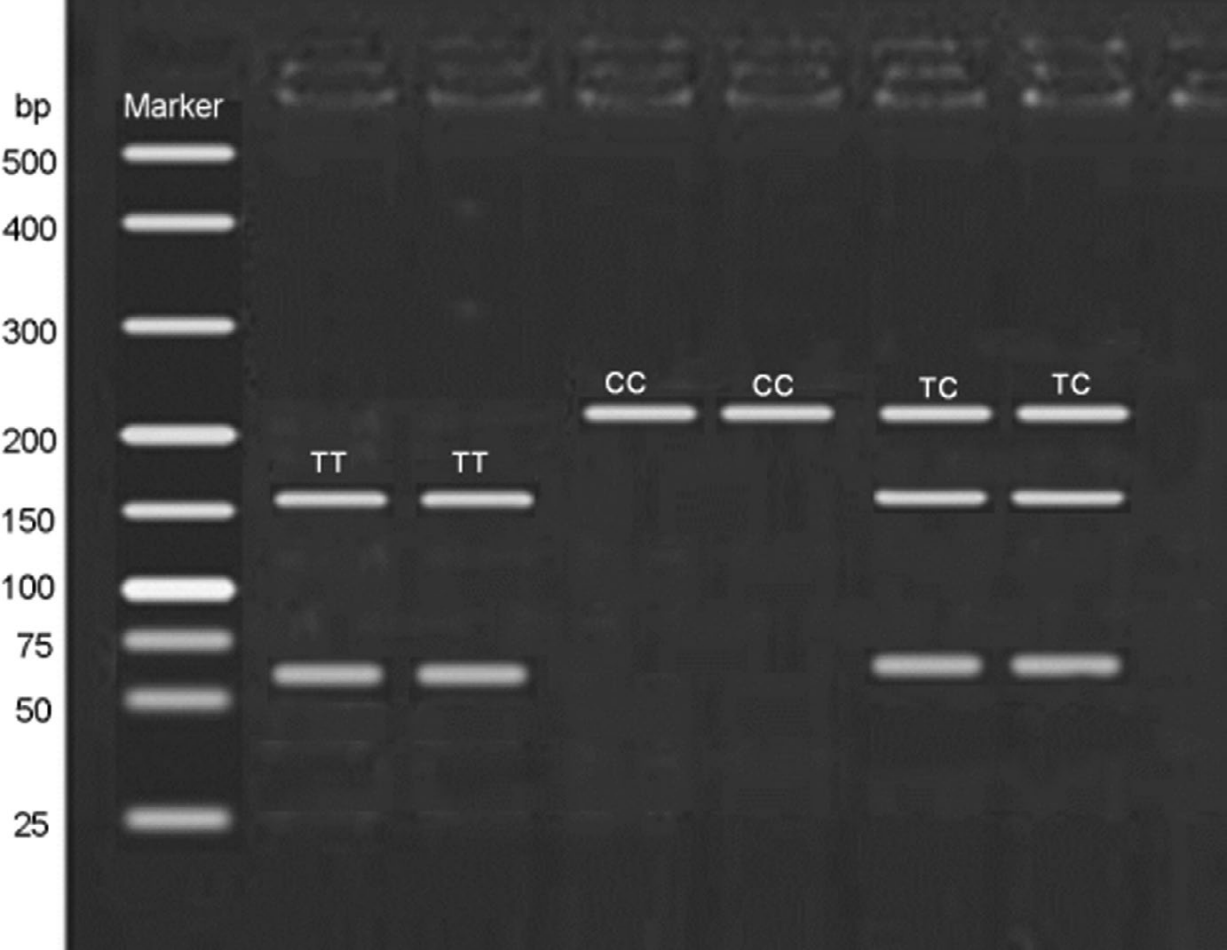
The digestion results for rs2069727 polymorphism. The amplified fragment of rs2069727 polymorphism was digested by *Hinf*I. In this setting, TT genotype had 159‐bp and 64‐bp fragments, CC showed 223‐bp fragment, and TC had 223‐bp, 159‐bp and 64‐bp fragments

*IFN‐γ* gene polymorphisms were genotyped by PCR‐RFLP method, and the results were in line with sequencing results. The amplified fragment of rs2069727 polymorphism was digested by *Hinf*I. As displayed in Figure 1, TT genotype had 159‐bp and 64‐bp fragments, CC showed 223‐bp fragment, and TC had 223‐bp, 159‐bp and 64‐bp fragments. The frequencies of rs2069727 TT, TC and CC genotypes were 56.48%, 32.41% and 11.11% in case group, and 70.00%, 27.27% and 2.73% in control group, respectively. The data showed that CC genotype of rs2069727 in *IFN‐γ* gene was associated with increased risk of AS (rough: OR = 5.049, 95% CI = 1.364‐18.694, *P* = 0.008; adjusted OR = 5.899, 95% CI = 1.563‐22.261, *P* = 0.009). Besides, the frequency of C allele also showed significant higher in case group than that in control group (27.31% vs 16.36%, *P* = 0.006). Chi‐square demonstrated that C allele of rs2069727 polymorphism was a risk factor for onset of AS (rough: OR = 1.921, 95% CI = 1.205‐3.061, *P* = 0.006; adjusted OR = 2.052, 95% CI = 1.286‐1.704, *P* = 0.003) (Table 2).

**TABLE 2:** Genotype and allele distributions of *IFN‐γ* gene rs2069727 and rs1861494 polymorphisms in case and control groups

Genotype/ Allele	Case, n = 108 (%)	Control, n = 110 (%)	*P*	OR(95% CI)	*P* ^a^	OR (95% CI)^a^
*rs2069727*						
TT	61 (56.48)	77 (70.00)	‐	Ref	‐	Ref
TC	35 (32.41)	30 (27.27)	.199	1.473 (0.815‐2.662)	.177	1.547 (0.827‐2.914)
CC	12 (11.11)	3 (2.73)	.008	5.049 (1.364‐18.694)	.009	5.899 (1.563‐22.261)
T	155 (72.69)	184 (83.64)	‐	Ref	‐	Ref
C	59 (27.31)	36 (16.36)	.006	1.921 (1.205‐3.061)	.003	2.052 (1.286‐1.704)
*P*_HWE_		0.970				
*rs1861494*						
CC	16 (14.81)	18 (16.36)	‐	Ref	‐	Ref
CT	46 (42.59)	45 (40.91)	.728	1.150 (0.522‐2.531)	.824	0.912 (0.405‐2.052)
TT	46 (42.59)	47 (42.73)	.810	1.101 (0.501‐2.418)	.816	0.906 (0.393‐3.273)
C	78 (36.11)	81 (36.82)	‐	Ref	‐	Ref
T	138 (63.89)	139 (63.18)	.878	1.031 (0.698‐1.523)	.894	1.027 (0.693‐1.522)
*P*_HWE_		0.205				

Abbreviations: ‐, indicated no related data; HWE, Hardy‐Weinberg equilibrium; Ref, reference.

^a^ The results were adjusted to age and gender using logistic regression analysis.

**FIGURE 2:**
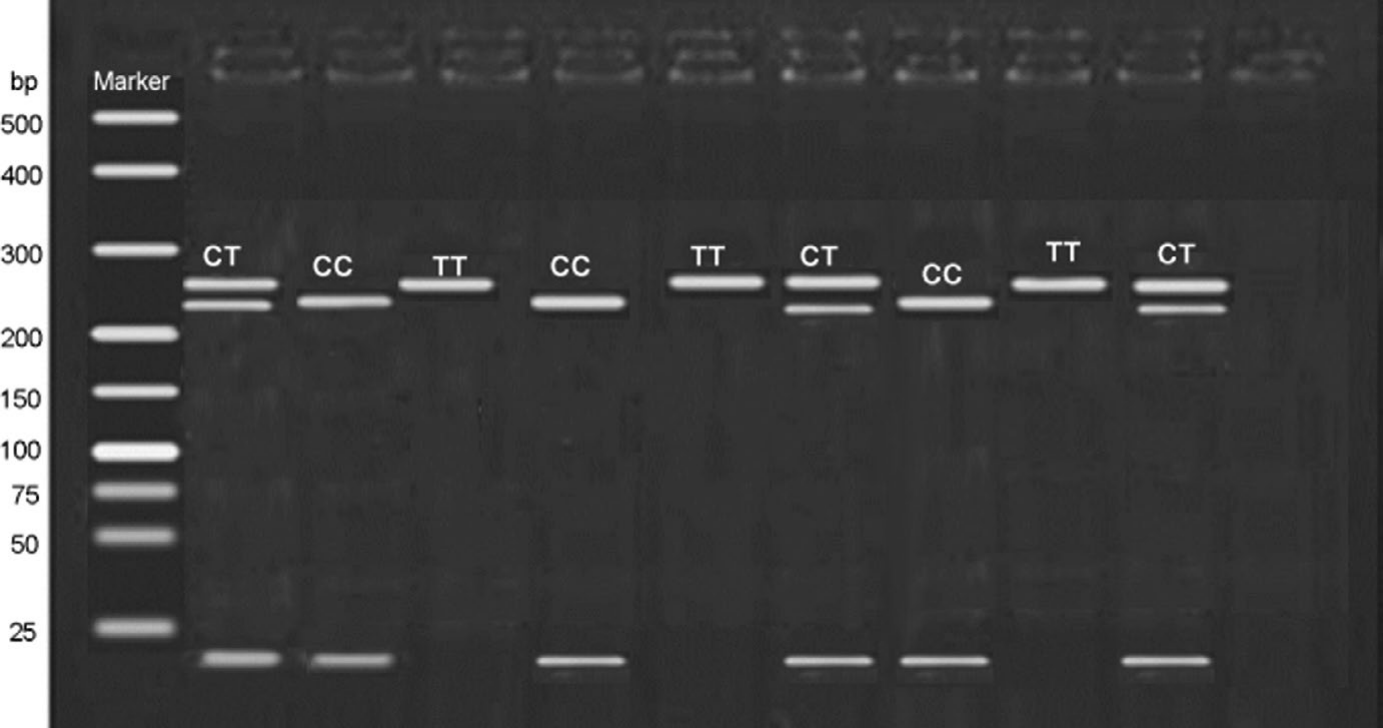
The digestion results for rs1861494 polymorphism. The amplified production of rs1861494 polymorphism was digested by *Msp*I, and the results were as follows: TT (267bp), CT (267bp, 245bp and 22bp) and CC (245bp and 22bp)

The amplified production of rs1861494 polymorphism was digested by *Msp*I, and the results were as follows: TT (267bp), CT (267bp, 245bp and 22bp) and CC (245bp and 22bp) (Figure 2). For rs1861494 polymorphism, frequencies of CC, CT and TT genotypes were 13.13%, 38.38%, 48.49% in AS patients, and 12.5%, 33.33%, 54.17% in controls respectively. C and T allele frequencies were 32.32% and 67.68% in cases, while the data in control group were 29.17% and 70.83%. However, no significant difference in the frequency of *IFN‐γ* rs1861494 genotype and allele distributions was observed between AS patients and healthy controls (*P > *0.05 for all) (Table 2).

## 4 DISCUSSION

AS is a chronic progressive inflammatory rheumatic disease which is regulated by multiple environmental and genetic factors, especially the inflammatory system components, like cytokines. As an anti‐inflammatory cytokine, *IFN‐γ* plays a modulatory role in the immune response. In this study, we investigated the genetic association of *IFN‐γ* rs2069727 and rs1861494 polymorphisms on risk of AS in Chinese Han population. Analysis results demonstrated that rs2069727 polymorphism showed close association with AS risk, but not rs1861494.

*IFN‐γ* belongs to type II class of interferons and plays crucial roles in multiple immune and inflammatory responses.^22^ Abnormalities in *IFN‐γ* expression may result in various inflammatory and immune diseases. It has been reported that gene expression and the activity of the corresponding protein may be regulated by its single nucleotide polymorphisms (SNPs).^23^ There are several polymorphisms in *IFN‐γ* gene, and some of them have been reported to be associated with activity of *IFN‐γ* gene, such as rs2430561.^24^ The genetic variants in *IFN‐γ* gene may be involved in human diseases via their regulatory roles in *IFN‐γ* production and immune response. *IFN‐γ* rs2069727 and rs1861494 are two commonly studied SNP, and their regulatory roles in transcriptional level of *IFN‐γ* gene have also been reported in the previous relevant studies.^18^, ^20^ However, to our knowledge, the genetic association of *IFN‐γ* rs2069727 and rs1861494 polymorphisms with AS risk has been rarely reported in Chinese Han population.

In current research, a case‐control study was designed to investigate the genetic effects of rs2069727 and rs1861494 polymorphisms in *IFN‐γ* gene on AS risk among Chinese Han population. Our findings supported that the individuals carrying rs2069727 CC genotype showed a significant higher risk to suffer from AS, compared with those carrying TT genotype. Similarly, rs2069727 C allele also showed increased risk to develop AS. The conclusion was in line with the previous studies. The study carried out by Li et al suggested that GG genotype of *IFN‐γ* rs2069727 polymorphism had apparently different distributions between case and control groups, and might confer increased risk of hepatocellular carcinoma.^25^ Alam et al reported the significant interaction of *IFNG* rs2069727 with gastrointestinal GVHD.^26^ In this study, we also examined the association between rs1861494 polymorphism and susceptibility to AS in the study population. Analysis results demonstrated that *IFN‐γ* gene rs1861494 polymorphism might have no obvious association with AS in Chinese Han population. The conclusion was partly in line with the studies carried in other inflammatory diseases. Gao et al reported that although *IFN‐γ* rs1861494 polymorphism could influence serum *IFN‐γ* level, there was no obvious association between the SNP with immunoglobulin A nephropathy (IgAN) in Chinese Han population.^27^ However, the study scheduled by Gonsky et al reported that *IFN‐γ* rs1861494 polymorphism could influence inflammatory bowel disease severity via its functional roles in *IFN‐γ* gene methylation and protein secretion among Caucasian population.^19^ The divergences might be attributed to the relative small sample size, different study populations and geographic regions, as well as the diverse aetiologies of the study diseases. Further multiple central studies will be required to investigate the genetic association of *IFN‐γ* rs1861494 polymorphism with AS.

Some limitations were found to disturb the veracity of our results. Firstly, the sample size was relatively small that reduced the statistical power of our analysis (less than 0.8). A further validation cohort study with enlarged sample size is required to verify and improve our results. Secondly, the gene‐gene and gene‐environment interactions, as well as the interactions between different SNPs of *IFN‐γ* gene, had not been taken in consideration. Additionally, the molecular mechanisms underlying the roles of *IFN‐γ* polymorphisms in aetiology of AS had not been explored in our study. Whether the detected polymorphisms were involved in pathogenesis of AS through regulating the transcriptional activity of the gene remained unclear. A well‐designed cohort study with a large sample size will be scheduled to address the above issues.

In conclusion, *IFN‐γ* rs2069727 SNP may contribute to risk of AS in the Chinese Han population, but not rs1861494 polymorphism.

## CONFLICT OF INTEREST

All authors declare that they have no conflict of interest.

## AUTHOR CONTRIBUTION

**Yanhui Liu:** Conceptualization (equal); Data curation (equal); Formal analysis (equal). **Guohui Zhang:** Conceptualization (equal); Data curation (equal). **Yulong Guan:** Formal analysis (equal); Funding acquisition (equal); Investigation (equal). **Xiaoliang Zhao:** Methodology (equal); Resources (equal); Software (equal). **Quan Wang:** Resources (equal); Writing‐review & editing (equal). **Hua Li:** Investigation (equal); Writing‐original draft (equal). **Jinhong Qi:** Methodology (equal); Writing‐review & editing (equal).

## Data Availability

All data generated or analysed during this study are included in this article.
